# Exposure to Greenness and Mortality in a Nationwide Prospective Cohort Study of Women

**DOI:** 10.1289/ehp.1510363

**Published:** 2016-04-14

**Authors:** Peter James, Jaime E. Hart, Rachel F. Banay, Francine Laden

**Affiliations:** 1 Department of Epidemiology, and; 2 Department of Environmental Health, Harvard T.H. Chan School of Public Health, Boston, Massachusetts, USA; 3 Channing Division of Network Medicine, Department of Medicine, Brigham and Women’s Hospital and Harvard Medical School, Boston, Massachusetts, USA

## Abstract

**Background:**

Green, natural environments may ameliorate adverse environmental exposures (e.g., air pollution, noise, and extreme heat), increase physical activity and social engagement, and lower stress.

**Objectives:**

We aimed to examine the prospective association between residential greenness and mortality.

**Methods:**

Using data from the U.S.-based Nurses’ Health Study prospective cohort, we defined cumulative average time-varying seasonal greenness surrounding each participant’s address using satellite imagery [Normalized Difference Vegetation Index (NDVI)]. We followed 108,630 women and observed 8,604 deaths between 2000 and 2008.

**Results:**

In models adjusted for mortality risk factors (age, race/ethnicity, smoking, and individual- and area-level socioeconomic status), women living in the highest quintile of cumulative average greenness (accounting for changes in residence during follow-up) in the 250-m area around their home had a 12% lower rate of all-cause nonaccidental mortality [95% confidence interval (CI); 0.82, 0.94] than those in the lowest quintile. The results were consistent for the 1,250-m area, although the relationship was slightly attenuated. These associations were strongest for respiratory and cancer mortality. The findings from a mediation analysis suggested that the association between greenness and mortality may be at least partly mediated by physical activity, particulate matter < 2.5 μm, social engagement, and depression.

**Conclusions:**

Higher levels of green vegetation were associated with decreased mortality. Policies to increase vegetation may provide opportunities for physical activity, reduce harmful exposures, increase social engagement, and improve mental health. Planting vegetation may mitigate the effects of climate change; in addition, evidence of an association between vegetation and lower mortality rates suggests it also might be used to improve health.

**Citation:**

James P, Hart JE, Banay RF, Laden F. 2016. Exposure to greenness and mortality in a nationwide prospective cohort study of women. Environ Health Perspect 124:1344–1352; http://dx.doi.org/10.1289/ehp.1510363

## Introduction

The biophilia hypothesis suggests that human beings have evolved to prefer certain natural environments that are essential to their thriving (Wilson 1984). Researchers are increasingly exploring how neighborhood greenness, or vegetation, may affect health behaviors and outcomes (Hartig et al. 2014; James et al. 2015a). Empirical research suggests that greenness may reduce obesity and promote physical activity (Lachowycz and Jones 2011) as well as improve cardiovascular health (Pereira et al. 2012), mental health (Alcock et al. 2014; Gascon et al. 2015), and birth outcomes (Hystad et al. 2014). Greenness has been hypothesized to benefit health by lowering exposure to air pollution, extreme heat, and noise; by increasing opportunities for physical activity; by providing a location for social engagement; and by decreasing psychological stress and depression through direct contact with nature (Hartig et al. 2014; Hystad et al. 2014; Taylor et al. 2015).

Evidence suggests that exposure to greenness may lower mortality rates, although many of these studies relied on aggregated data (Jonker et al. 2014; Lachowycz and Jones 2014; Richardson et al. 2010; Richardson and Mitchell 2010), which limit inferences about the effect of greenness on individual health. Many mortality studies relied on cross-sectional data and could not estimate exposure over time (Hu et al. 2008; Mitchell and Popham 2008), whereas others could not account for important potential confounding by race/ethnicity, individual-level smoking, and area-level socioeconomic factors such as median home value (Takano et al. 2002; Villeneuve et al. 2012). Some studies have observed contradictory findings. One ecological study conducted on the city level found that all-cause mortality was higher in greener cities (Richardson et al. 2012). An ecological analysis across the entire United Kingdom found that higher greenness was associated with lower cardiovascular and respiratory mortality among males; however, no significant associations were found among women (Richardson and Mitchell 2010). A recent analysis of greenness and mortality in male and female stroke survivors living in the Boston area found that greater exposure to greenness was associated with higher survival rates (Wilker et al. 2014). To our knowledge, no study has examined time-varying greenness and mortality in a nationwide prospective cohort of women while accounting for important potential confounding factors and addressing potential mediators. Our objective was to examine the association between greenness and all-cause mortality, as well as cause-specific mortality, in a large prospective study of women across the entire contiguous United States. We hypothesized that higher levels of surrounding greenness would be associated with lower rates of all-cause, cancer, respiratory, and cardiovascular mortality; that these associations would differ by mortality cause; and that findings would support mediation by air pollution, physical activity, social engagement, and mental health.

## Methods

### Population

**Figure 1:**
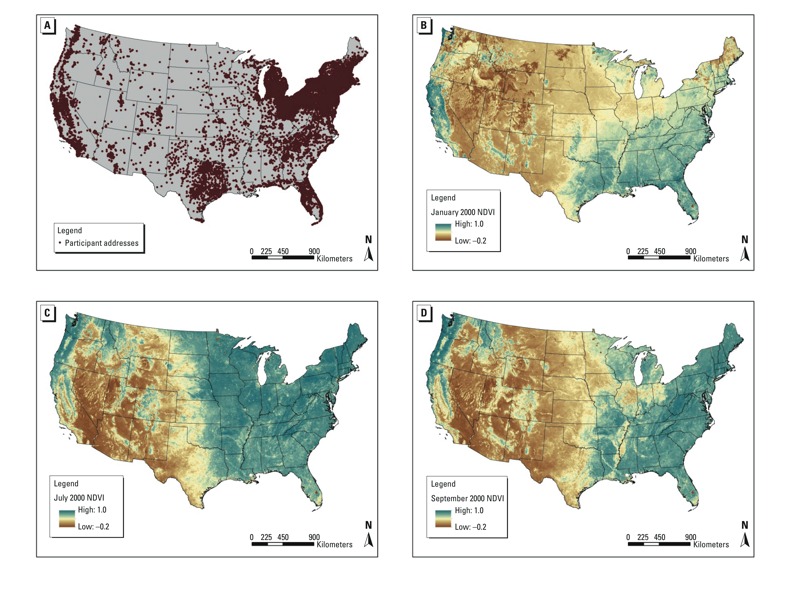
(*A*) Nurses’ Health Study geocoded residence locations at baseline (2000); NDVI 2000 Values in (*B*) January, (*C*) July, and (*D*) September. NDVI, Normalized Difference Vegetation Index.

The Nurses’ Health Study (NHS) is a prospective cohort study assessing risk factors for chronic disease among women. In 1976, 121,701 female registered nurses (30–55 years old) from 11 states (California, Connecticut, Florida, Maryland, Massachusetts, Michigan, New Jersey, New York, Ohio, Pennsylvania, and Texas) returned an initial questionnaire, which ascertained a variety of health-related exposures and medical diagnoses. The cohort has been continuously followed with biennial questionnaires. Response rates at each questionnaire cycle have consistently been ≥ 90% (calculated as the number of women who successfully returned a questionnaire in each cycle among the women who were still alive). Residential addresses from the 2000–2008 questionnaires were matched (geocoded) to obtain latitude and longitude. Approximately 90% of all addresses were successfully matched to the street segment level (within a range of house numbers along one side of the street). This analysis was conducted among all women who were alive in 2000 and had at least one residential address geocoded to the street segment level. Geocoded residence locations in 2000 are shown in Figure 1A; there were at least ten participating nurses in each of the contiguous United States. The study was approved by the Institutional Review Board of Brigham and Women’s Hospital, Boston, MA, and informed consent was implied through return of the questionnaires.

### Exposure

Exposure to vegetation around each participant’s home address was estimated using a satellite image–based vegetation index. Chlorophyll in plants absorbs visible light (0.4–0.7 μm) for use in photosynthesis, whereas leaves reflect near-infrared light (0.7–1.1 μm). The Normalized Difference Vegetation Index (NDVI) calculates the ratio of the difference between the near-infrared region and red reflectance to the sum of these two measures and ranges from –1.0 to 1.0, with larger values indicating higher levels of vegetative density (Kriegler et al. 1969). For this study, we used data from the Moderate-resolution Imaging Spectroradiometer (MODIS) from NASA’s Terra satellite. MODIS provides images every 16 days at a 250-m resolution (Carroll et al. 2004).

We used geographic information systems (GIS) software from ArcMap (ESRI, Redlands, CA) to estimate the mean NDVI value inside radii of 250- and 1,250-m buffers around each participant’s home. We chose the 250-m radius as a measure of greenness directly accessible outside each home and the 1,250-m radius as a measure of greenness within a 10- to 15-min walk based on prior work within the Nurses’ Health Study cohorts on neighborhood environments and health behaviors (James et al. 2014). We created a seasonally time-varying measure based on the NDVI for a representative month in each season (January, April, July, and October) (Figure 1B–D). Two exposure metrics were calculated for each radius: contemporaneous NDVI (the greenness value for the current season), to reflect short-term exposure to greenness, and cumulative average NDVI (updated based on changes in seasonal NDVI as well as on changes in address), to reflect long-term exposure to greenness. For both exposure metrics, exposures were updated as NDVI changed over time as well as when participants moved to new residential addresses (updated based on the receipt of a biennial questionnaire with a new residential address).

### Outcome

We assessed death occurring between the return of the 2000 questionnaire and 1 June 2008. Deaths were usually reported by families, and deaths among nonrespondents were identified by searching the National Death Index, which has been validated in prior studies in this cohort (Rich-Edwards et al. 1994). A physician reviewed death certificates and medical records to classify the primary cause of death according to the *International Classification of Diseases, Ninth Revision* (ICD-9). Our primary outcome was death from all nonaccidental causes. Secondary cause–specific analyses were conducted for the following categories (infectious and parasitic diseases, ICD-9 codes 0–139; cancer, ICD-9 codes 140–208; diabetes, ICD-9 code 250; neurodegenerative disease, ICD-9 codes 290, 332, 335, 340, 342, 348; coronary heart disease, ICD-9 codes 390–429, 440–459; stroke, ICD-9 codes 430–438; respiratory diseases, ICD-9 codes 460–519; kidney disease 580–593; and all other causes). Other deaths [including accidental (ICD-9 codes E800–E999)] were included as negative control outcomes to detect potential confounding bias (Lipsitch et al. 2010).

### Statistical Analysis

Person-months of follow-up were accrued from the return date of the 2000 questionnaire until either death or the end of follow-up (31 May 2008), whichever came first. We fit time-varying Cox proportional hazards models to compute hazard ratios (HRs) and 95% confidence intervals (CIs) for associations between each NDVI exposure measure and each mortality outcome. We examined the following covariates as potential confounders, effect modifiers, or mediators (all covariates time-varying unless otherwise indicated): fixed race/ethnicity (White non-Hispanic versus other), smoking status (current, former, never), pack-years of smoking, fixed individual-level socioeconomic status (SES), area-level SES, weight status {normal [body mass index (BMI) 18.5–24.9], overweight [BMI 25–29.9], obese [BMI > 30]}, region, urbanicity, whether a participant had changed addresses during follow-up, physical activity, air pollution, social engagement, and mental health. Current smoking status and pack-years smoked were updated at each biennial questionnaire. To account for fixed individual SES, we included information on self-reported parental occupation for the participant’s mother and father, whether the participant had a registered nursing degree, marital status, and husband’s highest educational attainment (< high school, high school graduate, > high school, missing or not married) as reported in 1992. We examined area-level SES by including information on census-tract median home value and census-tract median income based on the census tract containing the residential address at each questionnaire response and 2000 Census data (U.S. Census Bureau 2000). Urbanicity was determined by the participant’s residence in a metropolitan (urban area ≥ 50,000 people), micropolitan (urban cluster of 10,000–49,999 people), or small town/rural (urban cluster of < 10,000 people) census tract (Morrill et al. 1999). Physical activity was evaluated based on a validated biennial measure of self-reported total physical activity in the past year (Wolf et al. 1994). Although the specific activities varied on each questionnaire, questions included the average time per week spent walking, jogging (> 10 min per mile), running (≤ 10 min per mile), bicycling, lap swimming, playing tennis, playing squash or racquet ball, using a rowing machine, and engaging in calisthenics, aerobics, or aerobic dance. Each participant also reported the number of flights of stairs that she climbed daily and her usual walking pace. We multiplied the reported time spent weekly at each activity by its typical energy expenditure requirements expressed in metabolic equivalents (METs), then summed all the activity figures to yield a MET hours per week score and categorized total physical activity as < 3, 3–8.9, 9–17.9, 18–26.9, and ≥ 27 MET hr per week. These cut points were chosen to correspond to the equivalent of < 1, 1 to < 3, 3 to < 6, 6 to < 9, and ≥ 9 hr per week of walking at an average pace, consistent with prior analyses in this cohort (Colditz et al. 2003; Meyerhardt et al. 2006). Air pollution was quantified as quintiles of residential address-level 12-month average particulate matter < 2.5 μm in aerodynamic diameter (PM_2.5_) predicted from a spatiotemporal generalized additive mixed model (Yanosky et al. 2014). Social engagement was evaluated twice (2000 and 2004) over follow-up based on responses to the question “How many hours/week do you participate in groups?” (e.g., social, community, charity, etc.). We dichotomized social engagement based on whether participants reported participating in groups ≥ 1 hr per week. Mental health was based on biennial self-report of physician-diagnosed depression or regular antidepressant use.

Analyses were stratified by age of follow-up (months) and time period and were adjusted for race/ethnicity, smoking status, pack-years of smoking, and individual-level SES measures based on questionnaire responses, as well as on census tract area-level SES measures. We used the missing indicator method to account for missing covariate data. We used cubic regression splines to determine the linearity of exposure–response relationships (Durrleman and Simon 1989). Tests for nonlinearity used the likelihood ratio test, comparing the model with only the linear term to the model with the linear and the cubic spline terms. We report results for both continuous NDVI and NDVI quintiles. We examined the linear test for trend using the ordinal rank for each quintile. To test for violations of the proportional hazards assumption, we included interaction terms of each exposure and calendar time and performed likelihood ratio tests to determine statistically significant violations. Additionally, we tested for effect modification of the relationship between greenness and mortality by race/ethnicity, smoking, census tract median income, census tract median home value, weight status [defined by normal weight (BMI 18.5–24.9), overweight (BMI 25–29.9), and obese (BMI > 30)], physical activity, air pollution, region, urbanicity, and whether a participant had changed addresses during follow-up. We modeled interaction terms between continuous cumulative average NDVI in a 250-m buffer and each potential effect modifier and used likelihood ratio tests to assess statistical significance. We also obtained strata-specific effect estimates from stratified analyses. *p*-Values < 0.05 were used to define statistical significance. Data were analyzed using SAS 9.3 (SAS Institute Inc., Cary, NC).

We explored potential mechanisms through which greenness might affect mortality by evaluating the mediating effect of factors such as physical activity, air pollution exposure, social engagement, or mental health. We calculated the mediation proportion and its 95% CI using the publicly available %mediate macro (http://www.hsph.harvard.edu/donna-spiegelman/software/mediate/) (Lin et al. 1997). Briefly, the macro compares the exposure effect estimate from the full model that includes the exposure, one or more potential intermediate variables, and any covariates with the exposure effect estimate obtained from a partial model that leaves out the intermediate variable or variables. The mediation proportion is the proportion of reduced mortality explained by higher exposure to greenness that can be attributed to elevated levels of physical activity, air pollution exposure, social engagement, or mental health, as well as the joint effect of all of these mediators combined. Confidence intervals for the mediation proportion were calculated using the data duplication method (Lin et al. 1997). Mediation analyses assumed that there was no unmeasured exposure–outcome confounding, no unmeasured mediator–outcome confounding, no unmeasured exposure–mediator confounding, and no mediator–outcome confounder affected by exposure (VanderWeele 2015). Although these assumptions are unverifiable, we included major confounders in our mediation analyses, and therefore, we believe our assumptions are reasonable.

## Results

Participants were primarily White non-Hispanic, normal weight, and had low levels of physical activity (Table 1). The majority of participants lived in metropolitan areas, and half of the sample lived in the Northeastern United States. Those living in areas with higher levels of greenness were slightly younger, more likely to be White non-Hispanic, had husbands with higher levels of education, and lived in neighborhoods with higher SES. Areas with higher greenness had lower levels of air pollution.

**Table 1:** Age-adjusted Nurses’ Health Study participant characteristics by quintiles of cumulative average Normalized Difference Vegetation Index within 250-m buffers from 2000 to 2008 (*n* = 108,630).

Characteristic	Total	Greenness quintile 1	Greenness quintile 2	Greenness quintile 3	Greenness quintile 4	Greenness quintile 5
Cumulative average NDVI (250-m buffer) (mean ± SD)	0.47 ± 0.12	0.29 ± 0.08	0.42 ± 0.03	0.48 ± 0.03	0.54 ± 0.03	0.62 ± 0.05
Age (years)^*a*^ (mean ± SD)	68.98 ± 7.29	69.89 ± 7.26	69.42 ± 7.32	68.94 ± 7.30	68.49 ± 7.24	68.16 ± 7.18
White non-Hispanic (%)	94	90	94	94	95	95
BMI (Mean ± SD)	25.83 ± 7.35	25.33 ± 8.26	25.91 ± 7.43	26.08 ± 7.18	25.98 ± 7.08	25.82 ± 6.79
Weight status (%)
Normal weight (BMI 18.5–24.9)	39	38	38	38	39	41
Overweight (BMI 25–29.9)	33	32	33	33	33	33
Obese (BMI > 30)	23	23	23	24	23	21
Missing BMI	6	8	6	5	5	5
Total physical activity, MET hrs/week (%)
< 3	23	23	23	23	22	21
3 to < 9	21	20	21	22	21	20
9 to < 18	18	18	19	18	19	19
18 to < 27	11	11	11	11	11	12
≥ 27	19	18	19	19	19	21
Missing	8	11	8	7	7	7
Smoking status (%)
Never smoker	44	44	45	45	44	44
Former smoker	45	45	45	45	46	47
Current smoker	10	11	11	10	10	10
Have RN degree (%)	73	69	73	74	74	74
Married (%)	64	58	63	65	66	68
Husband’s highest education (%)
< High school	4	4	4	4	4	3
High school graduate	26	25	27	27	26	24
> High school education	35	32	33	34	36	41
Missing or not married	35	39	35	34	34	32
Census 2000–tract median income (mean ± SD, USD)	63,000 ± 24,000	57,000 ± 22,000	60,000 ± 22,000	62,000 ± 22,000	66,000 ± 24,000	72,000 ± 29,000
Census 2000–tract median home value (mean ± SD, USD)	170,000 ± 125,000	175,000 ± 138,000	159,000 ± 121,000	156,000 ± 115,000	167,000 ± 113,000	194,000 ± 133,000
12-month average PM_2.5_ (μg/m^3^) (mean ± SD)	12.03 ± 2.80	12.51 ± 3.74	12.23 ± 2.79	12.05 ± 2.45	11.88 ± 2.28	11.47 ± 2.38
Census-tract urbanicity (%)
Metropolitan (urban area ≥ 50,000 people)	84	86	83	83	84	83
Micropolitan (urban cluster of 10,000–49,999)	10	9	10	10	9	10
Small town or rural (urban cluster of < 10,000)	7	5	7	7	6	7
Region (%)
Northeast	50	35	42	52	63	56
Midwest	17	15	24	22	15	8
West	14	34	16	8	5	7
South	19	16	18	17	17	28
Moved during follow-up (%)	32	33	30	30	30	35
Depression (physician-diagnosed or antidepressant use) (%)	12	11	12	12	11	11
Social engagement (participation in groups > 1 hr per week) (%)	68	67	69	69	68	69
Abbreviations: BMI, body mass index; MET, metabolic equivalent of task; NDVI, Normalized Difference Vegetation Index; PM_2.5_, particulate matter < 2.5 μm in aerodynamic diameter; RN, registered nurse. ^***a***^Value is not age-adjusted.						

We observed 8,604 deaths over 627,008 person-years of follow-up among the 108,630 eligible cohort members. Table 2 shows HRs for the relationship between cumulative average greenness exposure and nonaccidental mortality. Analyses showed a consistent relationship between higher greenness and decreased mortality that was robust to adjustment for individual- and area-level covariates. In fully adjusted models, those living in the highest quintile of cumulative average greenness in the 250-m area around their home had a 12% lower rate of mortality (95% CI: 0.82, 0.94) than those in the lowest quintile. Results were consistent for the 1,250-m radius, although the relationship was slightly attenuated. Likelihood ratio tests from cubic regression spline analyses indicated that relationships between greenness and mortality rate were linear (data not shown). Continuous analyses also indicated an inverse association between greenness and mortality, with a 12% lower rate of mortality (95% CI: 0.82, 0.94) in fully-adjusted models based on a 0.1 increase in cumulative average NDVI in the 250-m area around participants’ homes. Again, the association was attenuated in the 1,250-m buffer. Results from models of contemporaneous NDVI were weaker but showed a generally consistent inverse association between greenness and mortality.

**Table 2:** Hazard ratios and 95% confidence intervals for greenness and nonaccidental*^a^* all-cause mortality in the Nurses’ Health Study (*n *= 108,630, with 8,604 deaths from 2000 to 2008).

Exposure metric	250-m buffer				1,250-m buffer			
	Cumulative average greenness		Contemporaneous greenness		Cumulative average greenness		Contemporaneous greenness	
	Age-adjusted HR (95% CI)	Fully adjusted HR (95% CI)^*b*^	Age-adjusted HR (95% CI)	Fully adjusted HR (95% CI)^*b*^	Age-adjusted HR (95% CI)	Fully adjusted HR (95% CI)^*b*^	Age-adjusted HR (95% CI)	Fully adjusted HR (95% CI)^*b*^
Quintile 1	Reference	Reference	Reference	Reference	Reference	Reference	Reference	Reference
Quintile 2	0.91 (0.85, 0.97)	0.92 (0.86, 0.98)	0.96 (0.90, 1.03)	0.96 (0.90, 1.02)	0.96 (0.90, 1.02)	0.95 (0.89, 1.01)	0.96 (0.90, 1.02)	0.95 (0.89, 1.01)
Quintile 3	0.88 (0.83, 0.94)	0.90 (0.84, 0.96)	0.93 (0.87, 0.99)	0.93 (0.87, 0.99)	0.95 (0.89, 1.01)	0.94 (0.88, 1.01)	0.95 (0.89, 1.01)	0.94 (0.88, 1.01)
Quintile 4	0.91 (0.85, 0.97)	0.94 (0.88, 1.00)	0.87 (0.82, 0.93)	0.89 (0.83, 0.95)	0.93 (0.87, 1.00)	0.94 (0.88, 1.01)	0.93 (0.87, 1.00)	0.94 (0.88, 1.01)
Quintile 5	0.83 (0.77, 0.88)	0.88 (0.82, 0.94)	0.89 (0.84, 0.96)	0.93 (0.87, 0.99)	0.86 (0.81, 0.92)	0.89 (0.83, 0.96)	0.86 (0.81, 0.92)	0.89 (0.83, 0.96)
*p* for trend^*c*^	< 0.0001	0.002	< 0.0001	0.003	< 0.0001	0.004	< 0.0001	0.004
Continuous (per 0.1 unit)	0.83 (0.78, 0.89)	0.88 (0.82, 0.94)	0.97 (0.94, 1.00)	0.99 (0.95, 1.02)	0.84 (0.79, 0.90)	0.89 (0.83, 0.95)	0.96 (0.93, 1.00)	0.98 (0.94, 1.01)
Abbreviations: CI, confidence interval; HR, hazard ratio. ^***a***^Excludes *International Classification of Diseases, Ninth Revision *(ICD-9) accidental codes E800–E999. ^***b***^Hazard ratios are adjusted for age and calendar year, race/ethnicity, smoking status, pack-years smoked, parental occupation, registered nurse (RN) degree, marital status, husband’s highest education, census-tract median home value, and census-tract median income. ^***c***^Based on linear test for trend using the ordinal rank for each quintile.								

Cause-specific mortality analyses revealed that the associations were strongest for respiratory, cancer, and kidney disease mortality, and we observed negative HRs for stroke mortality that were not statistically significant (Table 3). We estimated that those living in the highest quintile of cumulative average greenness in the 250-m area around their home had a 34% lower rate of respiratory disease–related mortality (95% CI: 0.52, 0.84), a 13% lower rate of cancer mortality (95% CI: 0.78, 0.97), and a 41% lower rate of kidney disease mortality (95% CI: 0.33, 1.05) than those in the lowest quintile. We did not observe any statistically significant associations between greenness and mortality from coronary heart disease, diabetes, or infections. In addition, associations for our negative control outcome of other deaths (including accidental, 185 out of the 1,219 other deaths) were null.

**Table 3:** Hazard ratios and 95% confidence intervals for cumulative average Normalized Difference Vegetation Index (250-m buffer) and cause-specific mortality in the Nurses’ Health Study (*n* = 108,630).

Outcome	Quintile 1	Quintile 2	Quintile 3	Quintile 4	Quintile 5	*p* for trend^*a*^	Continuous (per 0.1 unit)
Infectious and parasitic diseases (304 cases)
Cases	72	66	67	48	51
Adjusted HR (95% CI)^*b*^	Reference	0.99 (0.71, 1.40)	1.07 (0.76, 1.51)	0.82 (0.56, 1.19)	0.92 (0.63, 1.33)	0.411	0.78 (0.55, 1.10)
Cancer (3,363 cases)
Cases	778	695	654	650	586
Adjusted HR (95% CI)^*b*^	Reference	0.93 (0.84, 1.03)	0.90 (0.81, 1.00)	0.93 (0.83, 1.03)	0.87 (0.78, 0.97)	0.024	0.85 (0.76, 0.94)
Diabetes (145 cases)
Cases	37	32	20	33	23
Adjusted HR (95% CI)^*b*^	Reference	0.90 (0.56, 1.46)	0.59 (0.34, 1.02)	1.05 (0.65, 1.71)	0.81 (0.47, 1.38)	0.625	0.85 (0.52, 1.39)
Neurodegenerative diseases (827 cases)
Cases	188	173	173	146	147
Adjusted HR (95% CI)^*b*^	Reference	0.97 (0.78, 1.19)	1.03 (0.83, 1.27)	0.93 (0.74, 1.16)	0.98 (0.78, 1.22)	0.767	0.93 (0.75, 1.15)
Coronary heart disease (1,420 cases)
Cases	320	299	264	308	229
Adjusted HR (95% CI)^*b*^	Reference	0.99 (0.85, 1.16)	0.94 (0.79, 1.11)	1.19 (1.02, 1.40)	0.97 (0.81, 1.15)	0.474	1.02 (0.87, 1.20)
Stroke (606 cases)
Cases	155	114	140	109	88
Adjusted HR (95% CI)^*b*^	Reference	0.76 (0.59, 0.97)	0.99 (0.79, 1.26)	0.86 (0.67, 1.11)	0.77 (0.59, 1.01)	0.195	0.79 (0.62, 1.01)
Respiratory (766 cases)
Cases	213	163	158	129	103
Adjusted HR (95% CI)^*b*^	Reference	0.84 (0.69, 1.04)	0.86 (0.69, 1.06)	0.75 (0.60, 0.94)	0.66 (0.52, 0.84)	< 0.001	0.73 (0.59, 0.90)
Kidney (139 cases)
Cases	39	34	27	21	18
Adjusted HR (95% CI)^*b*^	Reference	0.93 (0.58, 1.48)	0.77 (0.47, 1.27)	0.64 (0.37, 1.11)	0.59 (0.33, 1.05)	0.029	0.63 (0.38, 1.04)
Other (1,219 cases)^*c*^
Cases	290	260	215	223	231
Adjusted HR (95% CI)^*b*^	Reference	0.95 (0.80, 1.12)	0.82 (0.69, 0.99)	0.91 (0.76, 1.09)	1.01 (0.84, 1.20)	0.810	1.01 (0.85, 1.20)
Abbreviations: CI, confidence interval; HR, hazard ratio. ^***a***^Based on linear test for trend using the ordinal rank for each quintile. ^***b***^Hazard ratios are adjusted for age and calendar year, race/ethnicity, smoking status, pack-years smoked, parental occupation, registered nurse (RN) degree, marital status, husband’s highest education, census-tract median home value, and census-tract median income. ^***c***^Includes any cause of death not included in the categories above, including accidental causes of death.							

### Stratified Analyses

**Figure 2:**
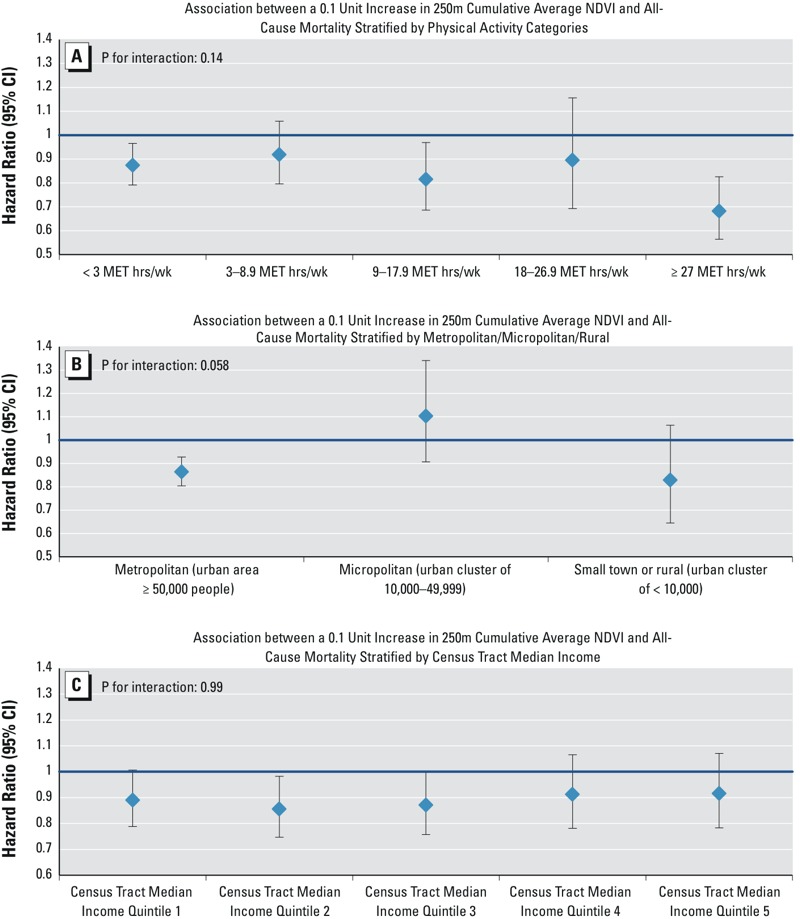
Hazard ratios and 95% confidence intervals for a 0.1-unit increase in cumulative average Normalized Difference Vegetation Index at the 250-m buffer and all-cause nonaccidental mortality in the Nurses’ Health Study (*n* = 108,630) stratified by (*A*) levels of physical activity, (*B*) census-tract metropolitan/rural or small town status, (*C*) census-tract median household income, and (*D*) predicted PM_2.5_ exposure. Hazard ratios are adjusted for age and calendar year, race/ethnicity, smoking status, pack-years smoked, parental occupation, registered nurse (RN) degree, marital status, husband’s highest education, census-tract median home value, and census-tract median income, except when stratifying variable. Abbreviations: MET, metabolic equivalent of task; NDVI, Normalized Difference Vegetation Index; PM_2.5_, particulate matter < 2.5 μm in aerodynamic diameter.

We observed no statistically significant differences in the association between greenness and mortality by race/ethnicity, smoking status, census tract median income, census tract median home value, PM_2.5_, weight status, region, urbanicity, or between movers and nonmovers (see Figure S1). Stratified analyses for physical activity, urbanicity, census tract median income, and PM_2.5_ are shown in Figure 2. We did observe a stronger association between greenness and mortality among participants with higher physical activity levels, although there was no statistical evidence of a difference in associations (*p* = 0.14). More precise relationships were observed for greenness in metropolitan areas compared with micropolitan and rural areas; however, we did not observe statistical evidence of a difference in associations across levels of urbanicity. This finding likely reflects that 84% of our sample lived in urban areas. There was no statistical evidence of a difference in associations across different levels of census tract median income (interaction *p*-value = 0.99).

### Mediation

Estimates of the proportion of the association between greenness and mortality that might be mediated by other factors (assuming that underlying assumptions of the mediation analyses hold) were statistically significant for physical activity, PM_2.5_, social engagement, and mental health (Table 4). The largest proportion of mediation was physician-diagnosed depression or antidepressant use, which was estimated to explain 30.6% (95% CI: 15.5%, 51.4%) of the association between cumulative average greenness in a 250-m buffer and mortality, and social engagement, which was estimated to explain 19.1% (95% CI: 10.0%, 33.3%) of this association. Although the estimated proportions explained were smaller, they were also statistically significant for physical activity [2.1% explained (95% CI: 0.2%, 19.3%)] and air pollution [4.4% explained (95% CI: 2.4%, 7.7%)]. The estimate from the joint mediation analysis suggested that all four mediators combined might explain 27.1% (95% CI: 14.7%, 44.6%) of the association between greenness and all-cause mortality. The findings were generally similar for greenness in a 1,250-m radius. Table S1 shows mediation results for cancer, respiratory, and kidney disease mortality, where we observed the strongest estimate of mediation by the mental health pathway and the weakest estimate of mediation by physical activity.

**Table 4:** Estimated proportion of association between greenness and mortality in the Nurses’ Health Study explained by physical activity, air pollution exposure, social engagement, and mental health*^a,b^*.

Mediator	Proportion of association of cumulative average greenness in 250-m buffer explained by mediator (95% CI)	Proportion of association of cumulative average greenness in 1,250-m buffer explained by mediator (95% CI)
Total physical activity (< 3 MET hr/week vs. ≥ 3 MET hr/week)	2.1% (0.2%, 19.3%)	1.1% (0.1%, 15.8%)
Air pollution [modeled PM_2.5_ < 9.7 μg/m^3^ (quintile 1) vs. ≥ 9.7 μg/m^3 ^(quintiles 2–5)]	4.4% (2.4%, 7.7%)	5.1% (2.4%, 10.5%)
Social engagement (participate in groups > 1 per week vs. ≤ 1 per week)	19.1% (10.0%, 33.3%)	12.8% (6.4%, 24.0%)
Mental health (physician-diagnosed or antidepressant use vs. none)	30.6% (15.5%, 51.4%)	25.5% (12.8%, 44.4%)
All mediators combined	27.1% (14.7%, 44.6%)	19.8% (10.2%, 35.0%)
Abbreviations: CI, confidence interval; MET, metabolic equivalent of task; PM_2.5_, particulate matter < 2.5 μm in aerodynamic diameter. ^***a***^Analyses adjusted for age and calendar year, race/ethnicity, smoking status, pack-years smoked, parental occupation, registered nurse (RN) degree, marital status, husband’s highest education, census-tract median home value, and census-tract median income. ^***b***^Mediation analyses assume that there is no unmeasured exposure–outcome confounding, no unmeasured mediator–outcome confounding, no unmeasured exposure–mediator confounding, and no mediator–outcome confounder affected by exposure.		

## Discussion

In this nationwide study of adult women, higher levels of greenness around each participant’s home address were associated with lower rates of all-cause, nonaccidental mortality regardless of adjustment for age, race/ethnicity, smoking status, individual-level SES, and area-level SES. These findings were strongest for cancer, respiratory, and kidney disease mortality. The results were consistent when focusing on the area immediately around each residence (250-m buffer) versus a larger radius (1,250-m buffer) around each participant’s home. The results were strongest when examining cumulative average exposure to greenness versus contemporaneous greenness, suggesting a larger health benefit of chronic exposure to greenness. The association between greenness and mortality was not statistically significantly different by race/ethnicity, physical activity, smoking status, area-level SES, air pollution exposure, weight status, region of the United States, whether a participant lived in a rural or urban area, or whether a participant moved during follow-up. Assuming that the assumptions of the mediation analysis hold, our estimates suggest that a large proportion of the association between greenness and mortality may be explained through mental health pathways of depression risk and social engagement, which subsequently affected mortality.

Our findings were consistent with, yet slightly stronger than, those of a general population mortality study that was cross-sectional (Mitchell and Popham 2008), as well those of as a study that was unable to adjust for individual-level smoking and sociodemographic characteristics (Villeneuve et al. 2012). Mitchell and Popham (2008) classified the percentage of green space for geographic units across England and observed lower levels of all-cause and circulatory mortality in the greenest areas, with an estimated 6% reduction (95% CI: 4%, 7%) in all-cause mortality in geographic units with the highest quintile of green space. A study of residents of Ontario, Canada found that an interquartile range (IQR) difference in NDVI (0.24) was associated with an estimated 5% (95% CI: 3%, 6%) reduced rate of nonaccidental mortality (Villeneuve et al. 2012). Wilker et al. (2014) followed patients in the Boston, Massachusetts area who had suffered ischemic strokes and found that those living in the highest quartile of NDVI had an estimated 22% (95% CI: 3%, 37%) lower rate of all-cause mortality than those in the lowest quartile. Our findings differed from those of an ecological study of 49 major cities across the United States that used the National Land Cover Database to define greenness (Richardson et al. 2012). This analysis showed that all-cause mortality rates were highest in the greenest cities; however, the city-level, cross-sectional analysis could not account for any individual-level factors, including smoking. In addition, an ecological cross-sectional study of urban wards across the United Kingdom found no relationship between greenness, measured through a land use database and satellite imagery, and cardiovascular, respiratory, or lung cancer mortality among women (Richardson and Mitchell 2010).

In cause-specific mortality analyses, we observed associations between greenness and respiratory, cancer, and kidney disease mortality. The findings for respiratory and cancer mortality are consistent with the pathway that greenness reduces air pollution exposure and increases physical activity, which are known to lower the risk of these outcomes (Hamra et al. 2014; Hoek et al. 2013; Lee et al. 2012). In addition, our findings on respiratory mortality were similar to those observed by Villeneuve et al. (2012). Although the absolute number of cases was small, there was a strong relationship between greenness and kidney disease that has not been observed in previous studies. This relationship could exist because greenness is linked to physical activity; inadequate physical activity is a risk factor for kidney disease (Stump 2011). We also observed suggestive evidence for a link between greenness and stroke mortality, which has been observed in other studies (Hu et al. 2008; Villeneuve et al. 2012). Although prior research has shown relationships between greenness and coronary heart disease, diabetes, and infections (Lachowycz and Jones 2014; Mitchell and Popham 2008; Rook 2013), we did not observe associations for these outcomes. A possible explanation for these discrepant findings is that our cohort of female nurses may utilize greenness in different ways than cohorts that include men (Richardson and Mitchell 2010) or individuals from other SES groups. Our finding of no association between greenness exposure and other deaths (including accidental), which can be considered negative controls because there is no clear mechanism for an association, lends confidence that the observed associations are not the result of uncontrolled confounding or other sources of bias that create a spurious causal inference (Lipsitch et al. 2010).

Our mediation analyses suggest that greenness affects all-cause mortality, as well as cancer, respiratory, and kidney disease mortality, through mental health, social engagement, physical activity, and air pollution. There is a foundation for each of these mechanisms in the literature. An analysis by de Vries et al. (2013) estimated that stress and social cohesion mediated the relationship between streetscape greenery and health. In their study, total physical activity was not a mediator; however, physical activity that took place in a public space did appear to mediate the greenery–health relationship. Higher exposure to greenness has been consistently linked to lower levels of depression, anxiety, and stress (Alcock et al. 2014; Beyer et al. 2014; Gascon et al. 2015). Studies have shown that views of nature may have a direct psychological benefit (Fuller et al. 2007). Higher levels of social engagement are also correlated with greenness exposure (Maas et al. 2009). Greenness appears to buffer exposure to air pollution (Dadvand et al. 2012; Su et al. 2009), and vegetation has been shown to remove particulate matter, sulfur dioxide (SO_2_), and carbon monoxide (Nowak et al. 2006), although evidence is inconsistent for other pollutants (Kim et al. 2013). Greenness may protect individuals from exposure to harmful noise (Gidlöf-Gunnarsson and Öhrström 2007) as well as alleviate thermal discomfort during heat stress (Lafortezza et al. 2009). Greater exposure to greenness has been associated with higher levels of physical activity (Almanza et al. 2012), but, consistent with our findings, other studies have shown that physical activity does not fully explain the relationship between greenness and health (Lachowycz and Jones 2014).

This study had a few limitations. The most appropriate scale at which to measure greenness is unclear (Mitchell et al. 2011). This uncertainty in the relevant geographic context to study exposure is a fundamental problem in spatial analyses (Kwan 2012). We explored two geographic scales (250-m and 1,250-m buffers) that yielded similar results, but it is unclear whether we would have observed stronger or weaker relationships if we had examined different scales. Although satellite-based measures of vegetation have been used extensively to measure exposure to greenness, NDVI does not measure the quality of greenness. Nevertheless, a validation study demonstrated that NDVI performs adequately when compared with environmental psychologists’ evaluations of green spaces (Rhew et al. 2011). Three environmental psychologists examined photographs, evaluated greenness on a Likert scale (none or very little to very high greenness), and compared their evaluations to NDVI measured in a 100-m radius around 124 homes in Baltimore, Maryland/Washington, DC and Seattle, Washington. Interrater reliability was high between environmental psychologist evaluations [intraclass correlation (ICC) = 0.82], and correlations between the photograph ratings and NDVI were high (*r* = 0.69, *p* < 0.001), indicating that NDVI may be a valid measure of greenness. Because of a lack of time-varying, nationwide data, we did not examine the association between exposure to major green spaces (e.g., parks) and mortality. The NHS data set enabled evaluation of many potential confounders and mediators; however, we remained limited in our ability to measure exposure to heat and noise, which could play important roles in how greenness affects health. In addition, the underlying assumptions required for estimates from the mediation analysis to be valid are unverifiable, which is a limitation of our mediation analysis. Selection into neighborhoods according to health status is a consistent concern in studies of geographic context and health. If participants in better health selected to move to neighborhoods with higher levels of greenness, this confounding by neighborhood preference could explain the relationship between greenness and mortality. However, in prior analyses, we showed that BMI and physical activity levels did not predict neighborhood selection by built-environment features in this cohort (James et al. 2015b); therefore, it is unlikely that neighborhood self-selection is a major concern. The consistent relationship between greenness and SES measures indicated the potential for strong confounding; however, we aimed to reduce the likelihood of confounding by adjusting for multiple individual- and area-level measures of SES. The association between greenness and mortality was consistent across different levels of area-level SES, which lends additional confidence that the observed relationships were not a consequence of unmeasured confounding by area-level SES. Additionally, the negative control outcome of other deaths was not associated with greenness, further decreasing the likelihood that our findings were an artifact of confounding. Because of the geographic distribution of our sample, it is likely that we had limited power to assess variation by region and by urban/rural differences. Finally, participants in this study were nurses at the time of recruitment, > 90% were White non-Hispanic, and all were female. Although the homogeneity of the study participants does restrict the generalizability of these results to the general population, it also eliminates confounding by sex and reduces the potential for confounding by SES and race/ethnicity.

This study also has a number of notable strengths. To our knowledge, it is the first prospective examination of the relationship between exposure to greenness and mortality across the entire United States. We were able to construct time-varying measures of exposure to greenness in the area surrounding each participant’s home address over 8 years of follow-up. Additionally, we were able to control for important confounders, such as smoking status and individual- and area-level SES. Because participants were located in a diverse range of geographic settings across the country, we were able to test whether the relationship between greenness and mortality was consistent in different regions, as well as in urban and rural locations. Finally, measurements of important intermediate exposures and behaviors enabled us to examine the potential mechanisms through which greenness affects mortality.

## Conclusions

In this nationwide cohort of adult women, we observed that those living in the highest quintile of satellite-measured green vegetation around their home had a lower mortality rate than those living in the lowest quintile of greenness. Findings were consistent across all regions of the United States, as well as in urban and rural areas, and we observed no threshold at which greater greenness exposure was not associated with lower mortality rates. Mediation analyses suggested that the association between greenness and mortality was explained primarily by improving mental health and increasing social engagement, as well as by lowering air pollution exposure and increasing physical activity. Although additional research is required on the relationship between other natural environments and health [e.g., blue spaces (Gascon et al. 2015)], these findings suggest that green vegetation has a protective effect and that policies to increase vegetation in both urban and rural areas may provide opportunities for physical activity, reduce harmful exposures, increase social engagement, and improve mental health. The recognized benefits of planting vegetation include reducing wastewater loads, sequestering carbon, and mitigating the effects of climate change (Jesdale et al. 2013); the additional evidence of an association between vegetation and reduced mortality rates suggests a potential co-benefit to improve health, presenting planners, landscape architects, and policy makers with an actionable tool to grow healthier places.

## Supplemental Material

(257 KB) PDF
