# Supplementary material for: Exposure to Greenness and Mortality in a Nationwide Prospective Cohort Study of Women
Source: Environ Health Perspect. 2016 Apr 14;124(9):1344–52. doi: 10.1289/ehp.1510363 (PMC5010419; doi:10.1289/ehp.1510363)
Supplement: (257 KB) PDF [file ehp.1510363.s001.acco.pdf]

Environ Health Perspect
DOI: 10.1289/ehp.1510363

Note to readers with disabilities: *EHP* strives to ensure that all journal content is accessible to all readers. However, some figures and Supplemental Material published in *EHP* articles may not conform to 508 standards due to the complexity of the information being presented. If you need assistance accessing journal content, please contact ehp508@niehs.nih.gov. Our staff will work with you to assess and meet your accessibility needs within 3 working days.

Supplemental Material

Exposure to Greenness and Mortality in a Nationwide Prospective Cohort Study of Women

Peter James, Jaime E. Hart, Rachel F. Banay, and Francine Laden

Table of Contents

Table S1. Estimated proportion of association between greenness and mortality in the Nurses' Health Study explained by physical activity, air pollution exposure, social engagement, and mental health

Figure S1. Hazard ratios and 95% confidence intervals for a 0.1 unit increase in cumulative average NDVI at the 250m buffer and all-cause non-accidental mortality in the Nurses' Health Study (N=108,630) stratified by a) Race/Ethnicity, b) Census region, c) Census tract median home value, d) $PM_{2.5}$ quintiles, e) Weight status, and f) Whether a participant moved over follow-up. Hazard ratios are adjusted for age and calendar year, race/ethnicity, smoking status, pack-years smoked, parental occupation, RN degree, marital status, husband's highest education, Census tract median home value, and Census tract median income, except when stratifying variable

Table S1. Estimated proportion of association between greenness and mortality in the Nurses' Health Study explained by physical activity, air pollution exposure, social engagement, and mental health[a, b]

**Mediator**	**Proportion of Association between Cumulative Average Greenness in 250m Buffer and Mortality Explained by Mediator (95% CI)**		
	**Cancer Mortality**	**Respiratory Mortality**	**Kidney-Disease Mortality**
**Total Physical Activity (metabolic equivalent of task-hrs/week)**	2.5% (0.7%, 9.1%)	Too small to calculate reliably (<1%)	Too small to calculate reliably (<1%)
**Air Pollution (Modeled $PM_{2.5}$)**	3.1% (1.3%, 6.9%)	2.1% (1.2%, 3.7%)	7.2% (2.5%, 18.9%)
**Social Engagement (Participate in Groups >1 per week)**	9.7% (4.2%, 21.1%)	4.9% (2.0%, 11.8%)	6.7% (2.4%, 17.3%)
**Mental Health (Doctor-Diagnosed or Antidepressant Use)**	19.9% (8.2%, 40.7%)	6.3% (2.1%, 17.1%)	9.6% (3.4%, 24.6%)
**All Mediators Combined**	15.3% (6.6%, 31.5%)	7.0% (3.1%, 14.9%)	11.2% (4.1%, 27.3%)

[a] Analyses adjusted for age and calendar year, race/ethnicity, smoking status, pack-years smoked, parental occupation, RN degree, marital status, husband's highest education, Census tract median home value, and Census tract median income

[b] Mediation analyses assume that there is no unmeasured exposure–outcome confounding; no unmeasured mediator–outcome confounding; no unmeasured exposure–mediator confounding; and no mediator–outcome confounder affected by exposure.

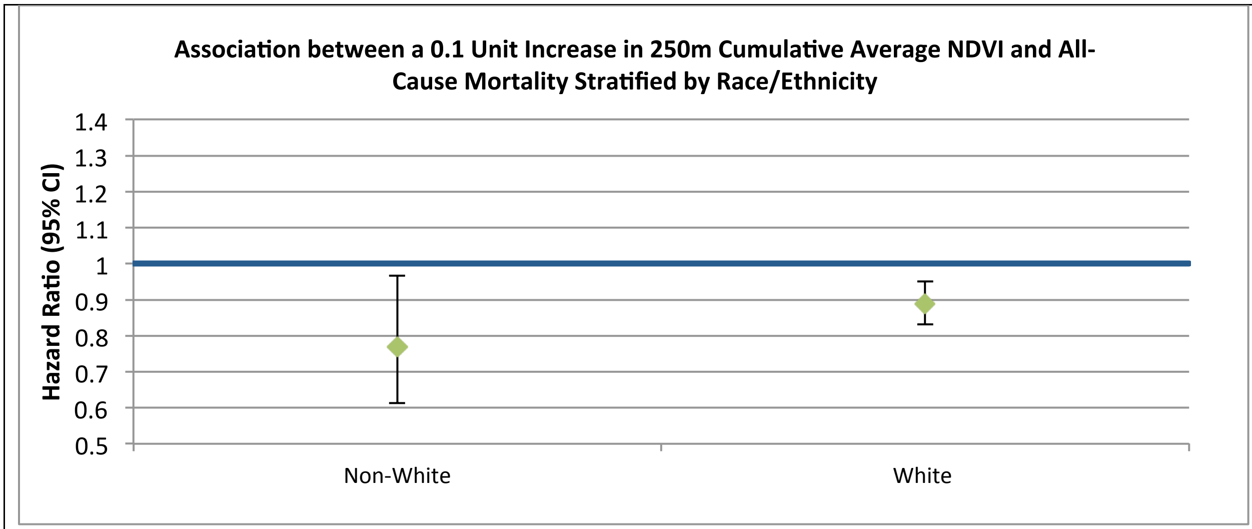

a) P for interaction: 0.23

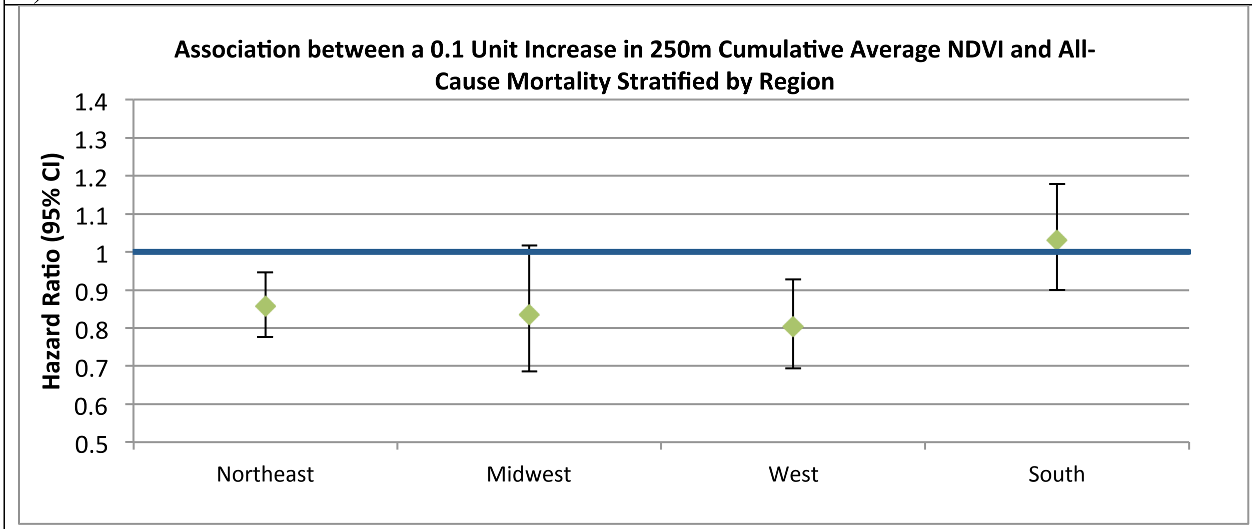

b) P for interaction: 0.061

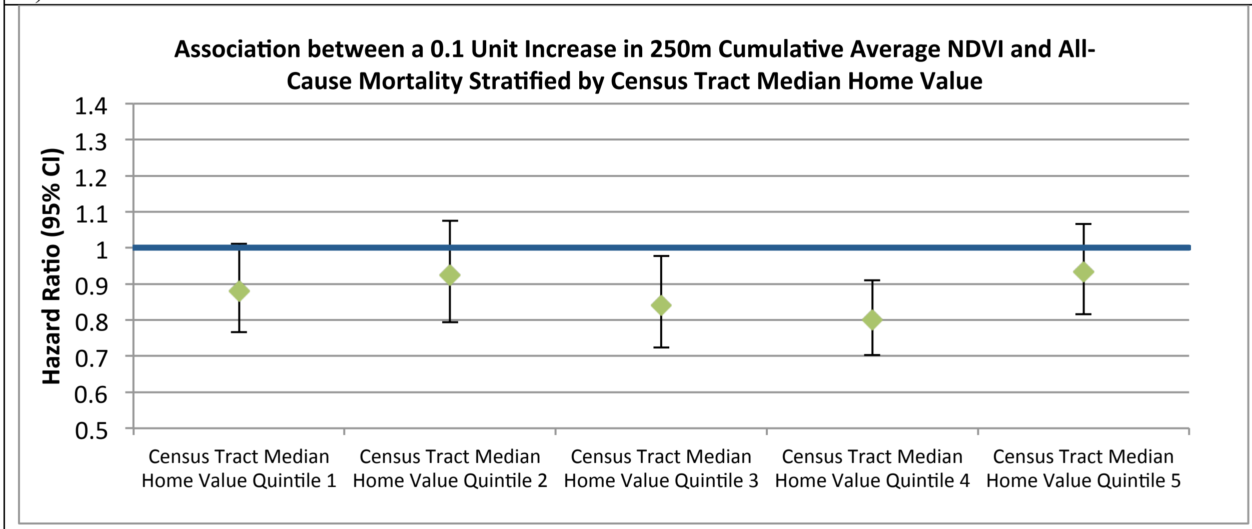

c) P for interaction: 0.61

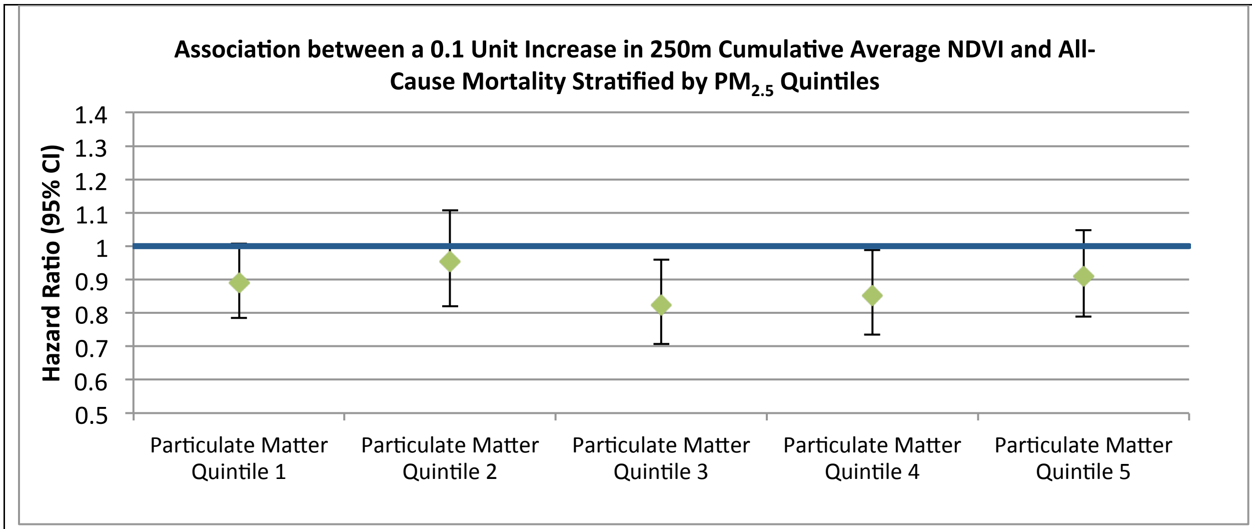

d) P for interaction: 0.69

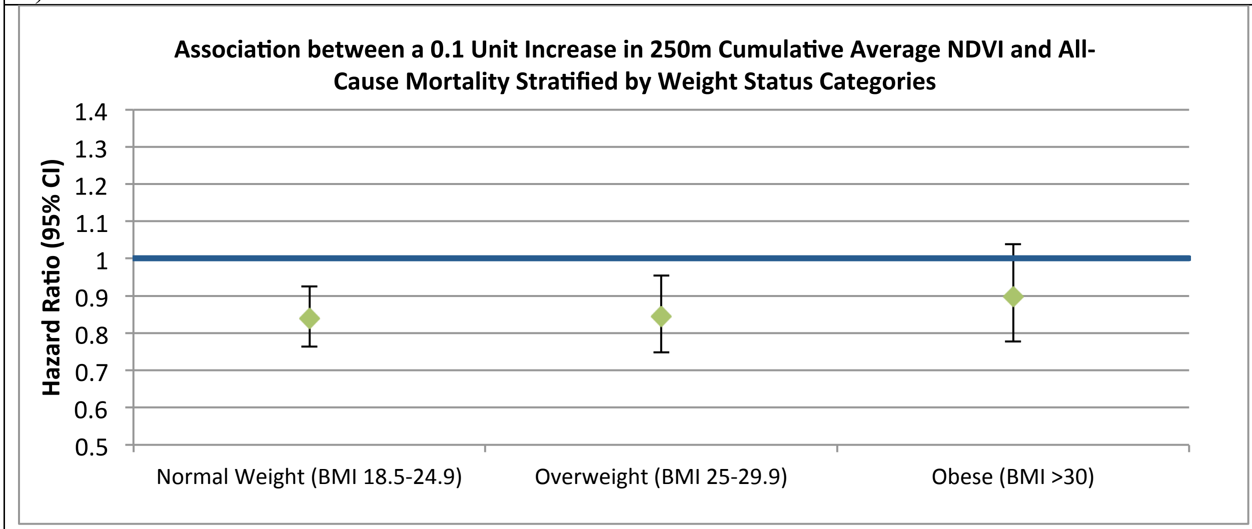

e) P for interaction: 0.73

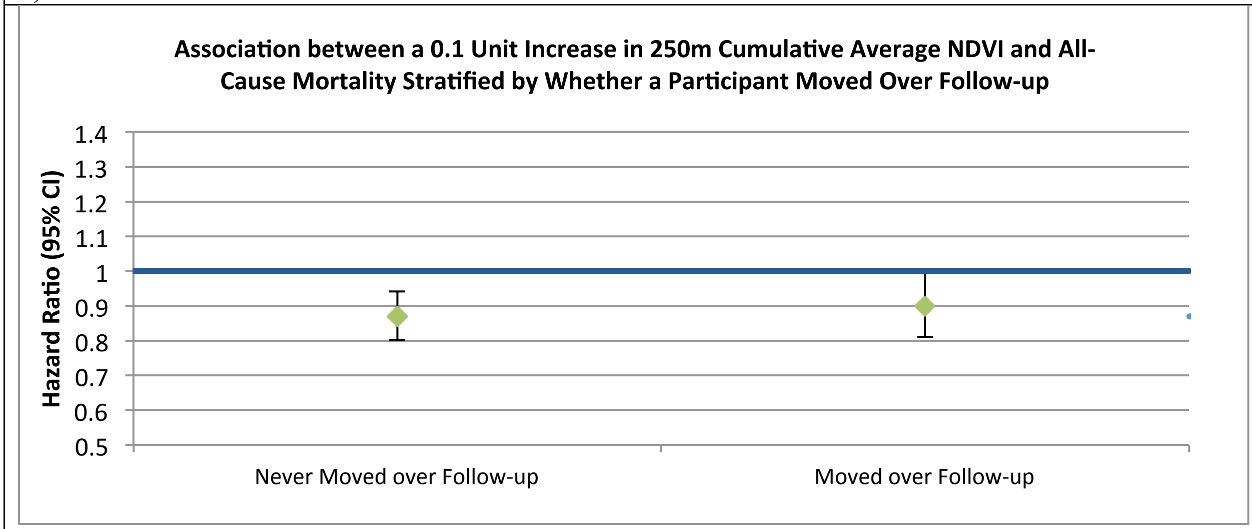

f) P for interaction: 0.59

Figure S1. Hazard ratios and 95% confidence intervals for a 0.1 unit increase in cumulative average NDVI at the 250m buffer and all-cause non-accidental mortality in the Nurses' Health Study (N=108,630) stratified by a) Race/Ethnicity, b) Census region, c) Census tract median home value, d) $PM_{2.5}$ quintiles, e) Weight status, and f) Whether a participant moved over follow-up. Hazard ratios are adjusted for age and calendar year, race/ethnicity, smoking status, pack-years smoked, parental occupation, RN degree, marital status, husband's highest education, Census tract median home value, and Census tract median income, except when stratifying variable
